# Measurement of emotional states of zebrafish through integrated analysis of motion and respiration using bioelectric signals

**DOI:** 10.1038/s41598-020-80578-6

**Published:** 2021-01-08

**Authors:** Zu Soh, Motoki Matsuno, Masayuki Yoshida, Akira Furui, Toshio Tsuji

**Affiliations:** 1 Graduate School of Advanced Science and Engineering, Hiroshima University, Higashi-Hiroshima, 739-8527 Japan; 2 Graduate School of Engineering, Hiroshima University, Higashi-Hiroshima, 739-8527 Japan; 3 Graduate School of Integrated Sciences for Life, Hiroshima University, Higashi-Hiroshima, 739-8527 Japan

**Keywords:** Biological techniques, Behavioural methods, Electrophysiology, Sensors and probes

## Abstract

Fear, anxiety, and preference in fish are generally evaluated by video-based behavioural analyses. We previously proposed a system that can measure bioelectrical signals, called ventilatory signals, using a 126-electrode array placed at the bottom of an aquarium and achieved cameraless real-time analysis of motion and ventilation. In this paper, we propose a method to evaluate the emotional state of fish by combining the motion and ventilatory indices obtained with the proposed system. In the experiments, fear/anxiety and appetitive behaviour were induced using alarm pheromone and ethanol, respectively. We also found that the emotional state of the zebrafish can be expressed on the principal component (PC) space extracted from the defined indices. The three emotional states were discriminated using a model-based machine learning method by feeding the PCs. Based on discrimination performed every 5 s, the F-score between the three emotional states were as follows: 0.84 for the normal state, 0.76 for the fear/anxiety state, and 0.59 for the appetitive behaviour. These results indicate the effectiveness of combining physiological and motional indices to discriminate the emotional states of zebrafish.

## Introduction

In the field of behavioural neuroscience, zebrafish have been used extensively as a model animal in behavioural experiments to study emotion expression mechanisms. Visual cues (viz., computer animation of the Indian leaf fish)^1^ and chemical substances, such as ibogaine^2^, have been used to evoke fear- and anxiety-related behaviour, and neural substrates related to fear have been examined using transgenesis^3^. Appetitive behaviours have been investigated using ethanol^4^ and caffeine^5^ in studies in which the zebrafish exhibited preferences for these substances. In these studies, video cameras were used to analyse the swimming velocity, absence of movement, and movement trajectory of zebrafish to quantify the behavioural responses. The data analysed in these studies were summarised in a behavioural catalogue that classifies approximately 200 types of behaviour patterns of zebrafish, including those associated with emotions such as fear, anxiety, and preference^6^.

Combining physiological states and motion could potentially provide deeper understanding or expand the detectable emotional states. For example, the behavioural catalogue^6^ indicates that the so-called freezing behaviour associated with anxiety is accompanied by rapid ventilation. Seeking to detect fear and anxiety responses without using a video tracking system, we previously proposed a system that can measure bioelectrical signals called ventilatory signals (see Supplementary Information) using a 126-electrode array placed at the bottom of an aquarium^7^. The system can simultaneously analyse ventilatory rhythm and motion to generate evaluation indices, and the resulted indices enabled the detection of fear and anxiety behaviours, such as zig-zag swimming and freezing, evoked by skin extract. However, the relationship between the index values obtained and appetitive behaviour has not been clarified. An index space that can detect appetitive behaviour could contribute to behavioural experiments by quantifying the emotions of the fish.

In this study, we evaluated the fear and anxiety evoked by the application of a skin extract containing an alarm pheromone and the appetitive behaviour evoked by the ethanol-induced place preference experiment using indices derived from measured motion and ventilatory signals.

## Materials and methods

### Real-time cameraless behavioural analysis system based on ventilatory signals

**Figure 1:**
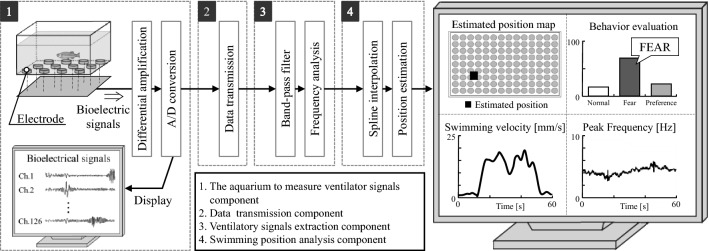
Structure of 2.1 real-time cameraless behavioral analysis system based on ventilatory signals (adapted from Ref.^7^). The system consists of an aquarium component for measuring ventilatory signals, a data transmission component, a ventilatory signals extraction component, and a component for estimating the position. The estimated position, the velocity, the peak frequency of the ventilatory signals, and an evaluation of the results are displayed on a PC monitor.

The structure of our previously proposed system is shown in Fig. 1. The system consists of an aquarium to measure ventilatory signals (see Supplementary Information), data transmission component, feature extraction component, and swimming position analysis component. The proposed system can analyse and measure ventilatory signals simultaneously in real-time at a sampling rate of 33 Hz, which is comparable to that of a general video analysis system. A comparison of behavioural analysis results obtained with the proposed system and with the video tracking system showed a low error level, with an average absolute position error of $9.75\pm 3.12$ mm (about one-third of the body length) and a correlation $r$ between swimming velocities of $0.93\pm 0.07 (p<0.01)$. The details of the system were described in a previous publication^7^. Here, we briefly describe the signal flow of the system.

1. Measurement of ventilatory signals
  The measurement aquarium was configured to be sufficiently large (210 (H) × 140 (W) × 65 (D) mm^3^) relative to the size of the zebrafish (approximately 35 mm). The ventilatory signals of the fish are measured under unconstrained conditions using an Ag–AgCl electrode array fixed to the bottom of the aquarium. An electroencephalograph (EEG-1200, Nihon Kohden Corp., Tokyo, Japan) is used to amplify the signals. An interface module (PCI-3521, Interface Corp., Hiroshima, Japan) is used for analogue-to-digital conversion of the signals measured by each of the 126 electrodes. The sampling frequency is ${f}_{s}$ =1000 Hz.
2. Data transmission
  Two PCs are used for real-time analysis: one to measure signals and the other to analyse motion. The signal measurement PC displays the ventilatory signals measured by each electrode. The data are transmitted to the motion analysis PC using a TCP/IP transmission protocol. The motion analysis PC receives ventilatory signal data and displays behavioural analysis results, such as the position and velocity of the fish.
3. Ventilatory signals extraction
  Ventilatory signals ${S}_{l}\left(n\right)(l=1,\dots ,L)$ are extracted by filtering the input signal with an ${M}_{b}$th-order bandpass filter with a low-range cutoff frequency ${f}_{low}=1$ Hz and high-range cutoff frequency ${f}_{high}=10$ Hz. The frequency spectrum of the signal ${S}_{l}$ is estimated using an autoregressive model^8,9^.
4. Swimming position analysis
  The position is estimated from the centre of gravity of the spatial distribution of the power spectral density peak calculated at a sampling frequency ${f}_{m}=33$ Hz. To calculate this spatial distribution, the distance between the electrodes was divided into five equal parts, and the spectral density peak is spline interpolated as a function of the distance between the electrodes. To reduce noise, the time series data for the centres of gravity are filtered with an ${M}_{lm}$ th-order low-pass filter with cutoff frequency ${f}_{cut1}$; the filtered coordinates are defined as the estimated position $\widehat{{\varvec{m}}}\left(n\right)=({\widehat{m}}_{x}\left(n\right),{\widehat{m}}_{y}\left(n\right))$ of the fish.

### Animals

Adult zebrafish Danio rerio with AB genetic background, originally established by Streisinger and Walker^10^ (The Zebrafish Information Network), and age between 12 and 18 months (sex not determined) were used for both extraction of alarm pheromone and behavioural experiments. The fish were reared in our laboratory and housed in groups of 5–7 in 2 L tanks on a 14:10 light and dark cycle. The water temperature was kept between 26 and 28 °C. The temperature of the room for measurements was maintained at 28 °C to keep the water temperature of the experimental aquarium at approximately 27 °C.

All of the experiments were conducted in accordance with the guidelines for animal experimentation of Hiroshima University. All animal experiments were carried out using procedures approved by the Committee on Animal Experimentation of Hiroshima University, Japan (approval number F15-1).

### Alarm pheromone experiment

Fear and anxiety can be induced artificially using an alarm pheromone contained in the epidermis of fish^11^. In the present study, we obtained an extract containing an alarm pheromone through the following process.

1. Deeply anaesthetised zebrafish were euthanised by cutting the medulla–spinal boundary, and the scales were peeled off the trunk.
2. The scales with the epidermis attached were ground in a small amount of distilled water, and the supernatant containing the epidermis extract was collected.
3. The collected epidermis extract, or alarm pheromone solution, was diluted by distilled water to obtain the solution containing the extract from one fish in 1 mL. Then, aliquots of 0.1 mL diluted solution were dispensed in cryotubes and stored at − 20 °C until use^12^.

On the day of the experiments, the alarm pheromone solution was thawed and diluted to 50 mL with distilled water and stored in a fridge. A 5 mL aliquot of the solution, which contained the alarm pheromone extract obtained from 1/100 individual, was used for the behavioural experiment after leaving it for 45 min at room temperature. The concentration of the alarm pheromone solution was determined using the procedure described in the previous report^13^, and pilot experiments to induce marked responses in zebrafish were conducted.

**Figure 2:**
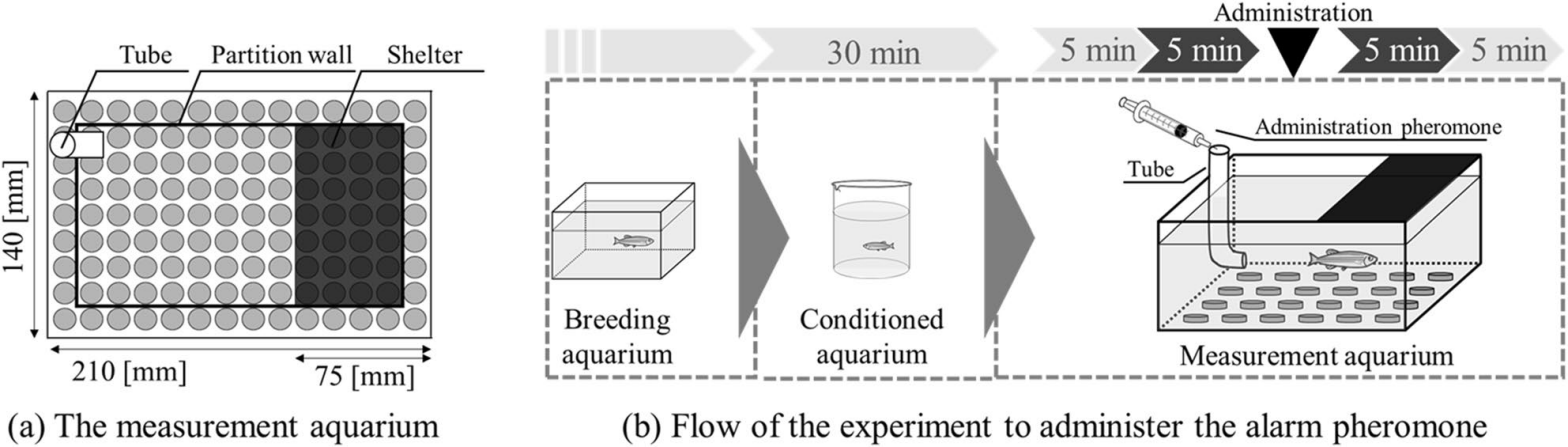
Experimental protocol for exposing the fish to an alarm pheromone. A shelter for hiding and a tube for introducing the substance are installed in the aquarium.

Figure 2 shows the experimental protocol followed for evaluating fear and anxiety behaviours. Figure 2a shows the tube used for exposing the alarm pheromone and a shelter fixed to the measurement aquarium. The shelter (75(W) × 140(D) mm^2^) is sufficiently large to allow a fish to swim and hide and provides a dark environment. To diffuse the alarm pheromone throughout the entire aquarium, the tube was installed at the bottom corner of the aquarium, outside the shelter. Because capturing zebrafish from a home tank could affect the later responses of the fish, the fish were first placed in an acclimatisation aquarium for 30 min (see Fig. 2b). Then, the fish were carefully poured into the measurement aquarium in water from the acclimatisation aquarium. The measurements were carried out for a total of 20 min. The fish were exposed to the alarm pheromone after spending 10 min in the measurement aquarium. Therefore, the behaviour shown in the first 10 min and the second 10 min can be considered to represent the normal state and fear/anxiety state, respectively. Observations were made 5 min before and 5 min after exposure to the alarm pheromone. We did not evaluate the first 5 min to exclude the effects caused by transferring the fish from the conditioned aquarium. The data of the last 5 min were discarded to equalise the evaluation duration before and after the administration of the alarm pheromone. To eliminate the influence of the water temperature on the response of the fish, the water in all aquariums was maintained at the same (room) temperature. After each experiment, the measurement aquarium was cleaned with a medical detergent to wash out the alarm pheromone. The experiments were conducted with fifteen zebrafish (Fish 1–Fish 15).

### Ethanol-induced place preference experiment

Previous studies^4^ reported that zebrafish exhibit a preference for ethanol. Fish exposed to ethanol in a specific compartment of an aquarium exhibit place preference in that the fish will stay in the compartment even if ethanol is not subsequently presented. It has been suggested that this behaviour is caused by the expectation for re-intake of ethanol, which can be interpreted as a type of appetitive behaviour^4^. In this study, we used ethanol to induce appetitive behaviour with expectation.

**Figure 3:**
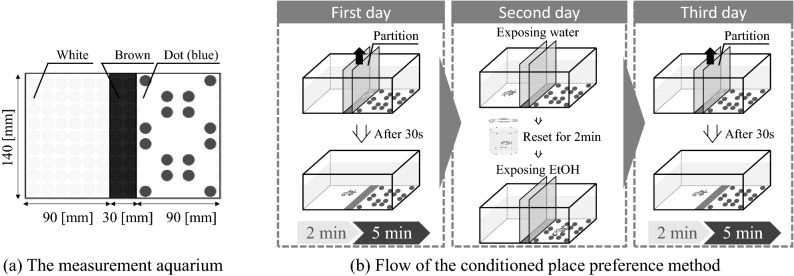
Experimental protocol for exposure of fish to ethanol. The experimental environment was configured based on the literature^4^. (**a**) Measurement aquarium, divided into three sections using gauze with different colour patterns. (**b**) Flow chart of the place preference conditioning protocol. The initial place preference is measured on the first day. The fish is conditioned on the second day to induce a place preference. The ventilatory signals and motion on the first and third day were analysed and compared.

Figure 3a shows the configuration of the measurement aquarium^4^. The measurement aquarium is divided into three sections using gauzes, painted white, brown, and with dots, which are laid on the bottom. The area ratio is white:brown:dot = 3:1:3 (see Fig. 3a). The gauze with the dot pattern has 16 dots with diameters of 10 mm. The centre section with the brown gauze on the bottom can be separated from the other sections by two partitions.

Figure 3b shows the experimental protocol for evaluating behaviour associated with ethanol-induced place preference. On the first day, a fish was put into the centre section, with partitions that separated it from the other sections. After 30 s, the partitions were removed (see Fig. 3b) so that the fish could swim freely within the measurement aquarium. After 2 min, the initial place preference of the fish was measured for 5 min. On the second day, the fish was exposed to ethanol in the section in which it did not exhibit a place preference and to dechlorinated water in the section in which it did exhibit a preference, for 20 min each. These procedures were performed in random order to eliminate any order effect. On the third day, the fish's final place preference was measured using the same procedure as that used on the first day. The behaviours exhibited on day 1 and day 3 were regarded as the fish's normal state and appetitive state, respectively. Data collected for 5 min from immediately after removing the partition on the first day and on the third day were analysed. When the fish stayed much longer in one section than in the other section on the first day, it was considered that the fish showed a strong initial preference. From preliminary experiments, we found that these fish were difficult to condition. Therefore, if the fish stayed for more than 80% of the measurement time in a section on the first day, further experiments were not conducted. The temperature of the water was maintained at room temperature and was cleaned with a medical detergent after every experiment. Experiments were conducted on 18 zebrafish (Fish 16–Fish 33). The data for eight of these fish (Fish 16–Fish 23) for which a place preference was successfully induced were analysed.

### Evaluation indices

This section describes the indices extracted to evaluate emotional states based on biological knowledge explained in the behavioural catalogue^6^. The indices were extracted as follows.

(i) Swimming velocity
  Fear and anxiety behaviour in zebrafish includes zig-zagging with multiple darting motions. Therefore, the swimming velocity $\widehat{{\varvec{v}}}\left(n\right)=({\widehat{v}}_{x}\left(n\right),{\widehat{v}}_{y}\left(n\right))$ is estimated from the time differential of the estimated position. To reduce noise due to position estimation errors, an ${M}_{ls}$th-order low-pass filter with a cutoff frequency of ${f}_{cut2}$ Hz was applied for the time-differentiated coordinates.
(ii) Probability of stopping
  After zig-zagging, the zebrafish exhibits freezing behaviour, accompanied by frequent gill movement. The stopping probability ${P}_{\rm{stop}}\left(n\right)$ was calculated as follows:
  1 $$P_{stop}(n) = \frac{T(|\hat{\varvec{v}}(n)|<v_{stop})}{T_{{index}}}\times100,$$
  where $T(\widehat{v}\left(n\right)<{v}_{stop})$ is considered to be stopping duration, during which the swimming velocity is below a threshold velocity ${v}_{stop}$, and ${T}_{index}$ is the analysis time window.
(iii) Ventilatory signal frequency
  To evaluate the frequency of gill movement, the ventilatory signal frequency ${F}_{v}\left(n\right)$ Hz was defined as the peak frequency at the estimated position.
  Finally, to evaluate the changes in indices over a short period of time, the standard deviations (SD) of each index were calculated, which yielded values for the following three indices.
(iv) SD of the swimming velocity
(v) SD of the stopping probability
(vi) SD of the ventilatory signal frequency

In total, values for six evaluation indices were calculated.

### Statistical analysis of the indices

The indices (i)–(iii) were calculated at 33 Hz and averaged every 5 s, and the SDs of these indices that corresponding to the indices (iv)–(vi) were calculated for the same 5 s period. Because the analysis lasted 5 min, the data size of each individual was 60 for each treatment. For the alarm pheromone experiment, each index was standardised by setting the mean and SD of the index values collected 5 min before exposure to 0 and 1, respectively. Then, we performed Welch's *t*-test to compare the indices between before and after the administration of alarm pheromone. The same procedure was performed on the ethanol-induced place preference experiment. The standardisation was performed using the index values of the first day, and the Welch’s *t*-test was carried out to compare the indices between the first and third days. *p* < 0.001 was considered to be significant.

### Discrimination of emotional state

We performed PC analysis (PCA) on the six evaluation indices^14^ collected from all individuals. Then, we visualised the distribution of the first and second PC scores of the three emotional states—the normal state, fear/anxiety state, and appetitive state, using a scatter plot.

Using Fisher's linear discriminant analysis^15^, a model was constructed to distinguish the normal state ${C}_{1}$, fear/anxiety state ${C}_{2}$, and appetitive state ${C}_{3}$. Linear discriminant analysis for classification is based on the following equations:

2 $$y_k(\varvec{x})=\varvec{w}_k^T\varvec{x},$$

3 $$\forall j \ne k, y_k(\varvec{x})>y_j(\varvec{x})\to\varvec{x}\in C_k,$$

where ${y}_{k}({\varvec{x}})$ and ${y}_{j}({\varvec{x}})$ are discriminant functions of emotional states *C*_*k*_ and *C*_*i*_, respectively, ${\varvec{x}}\in {\mathfrak{R}}^{N}$ is an input vector whose elements are the first through *N*th PC scores, and ${{\varvec{w}}}_{k}$ is a weight vector. Using a distribution of the evaluation indices of the normal, fear/anxiety, and appetitive state, the ratio of the variance between the three classes is maximised, and the variance within each class is minimised. Then, the coefficient of the discriminant function is determined; this function enables discrimination between fear, anxiety, and appetitive states from the defined indices.

To consider the nonlinear relationship between the emotional state and the defined indices, a probabilistic neural network called a log-linearised Gaussian mixture network (LLGMN)^16^ was used to discriminate the emotional states (see Supplementary Information). The LLGMN consists of a Gaussian mixture model and a log-linearised model. By learning the probability distribution of sample data, the LLGMN can estimate the posterior probability of each emotional state for a set of input index values. For both discrimination methods, we performed leave-one-fish-out cross-validation to obtain the discrimination performance. Discrimination was performed using the first through *N*th PC scores at 5 s intervals for each fish. The discrimination performance of the methods was then evaluated based on the F-score, which is the harmonic mean of precision and recall; this enables the balanced evaluation on the unequal fish number used in the alarm pheromone experiment and ethanol-induced place preference experiments. The number of the PC *N* was added one by one to test the significance of each PC in discrimination performance. The F-score obtained from the different PC numbers was compared using the Tukey–Kramer test. *p* < 0.001 was considered to be significant.

## Results

### Alarm pheromone experiment

**Figure 4:**
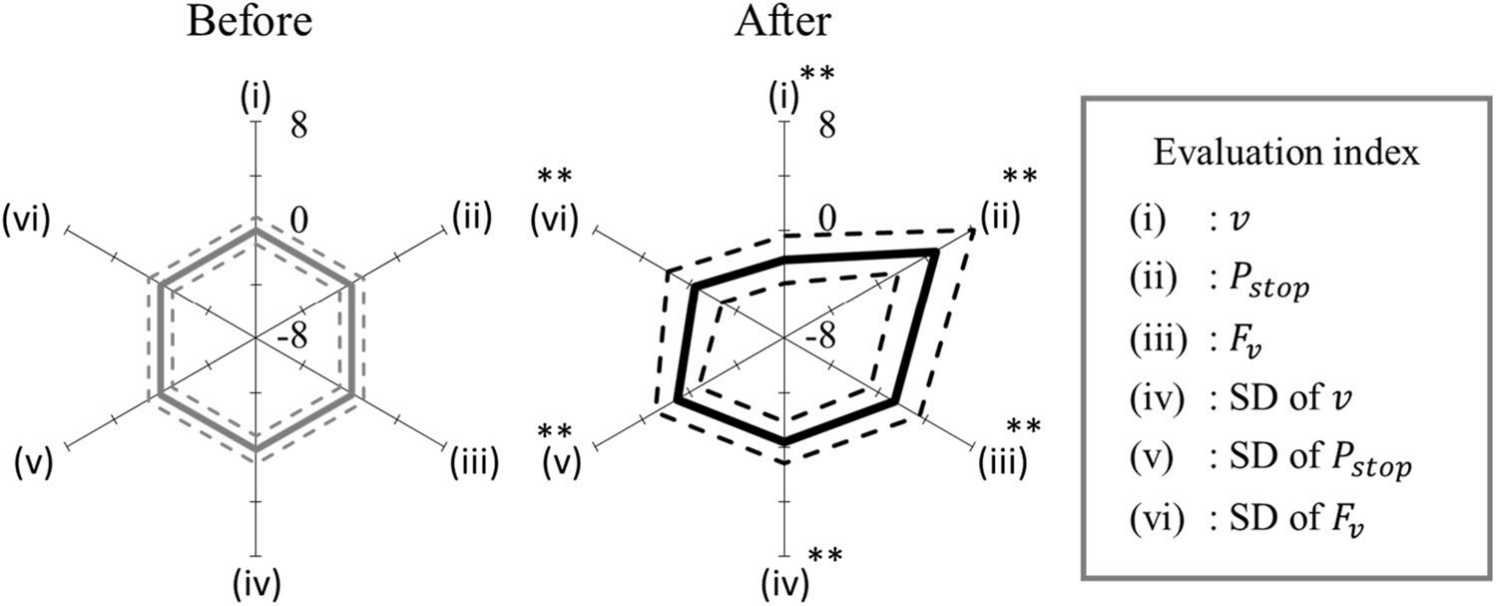
Radar chart for the evaluation indices before and after alarm pheromone exposure. The results indicate that the evaluation index values changed drastically after the fish were exposed to the alarm pheromone.

Figure 4 shows a radar chart of the evaluation indices before and after exposure to the alarm pheromone. Exposure to the alarm pheromone significantly lowered the swimming velocity $$(p<0.001$$), increased the stopping probability ($$p<0.001)$$, increased the ventilatory frequency ($$p<0.001$$), decreased the SD of the swimming velocity $$(p<0.001$$), ventilatory frequency ($$p<0.001$$), and increased the SD of the stopping probability ($$p<0.001$$). These responses were due to the freezing behaviour, i.e. staying still on the bottom with a high respiration rate evoking a fear/anxiety state.

### Ethanol-induced place preference experiment

**Figure 5:**
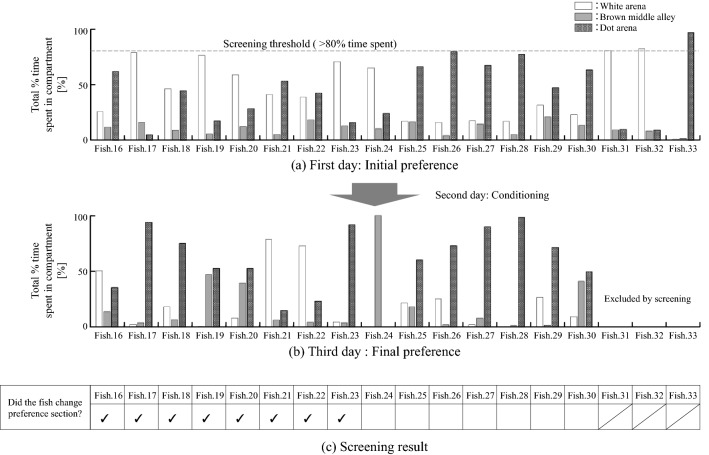
Changes in section preference. For Fish 16 to Fish 23, the preferred section on the third day was different from that on the first day.

**Figure 6:**
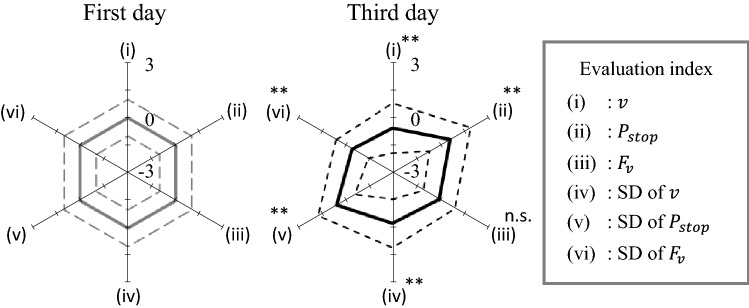
Radar chart for the evaluation indices before and after ethanol exposure.

Figure 5 shows the preference change from the first day to the third day. Eight fish (Fish 16–23) changed preference sections (see Fig. 5a,b). Experiments after the second day were not performed on Fish 31–Fish 33 because the time during which they stayed in one section on the first day exceeded 80%. Figure 6 shows the radar chart of the evaluation indices on the first and third days for eight individuals (Fish 16–Fish 23). Significant differences were found in all indices except for the ventilatory frequency between the first day and the third day; the *p*-values for all indices were $p<0.001$ except for the ventilatory frequency ($p=0.06$). The results show that on the third day, the variation in the index values for most of the fish were higher but the SD of the respiratory frequency was significantly lower. These results indicate that the appetitive state induces various behaviours and stabilises the rhythm of respiration.

### Analysis and discrimination of emotional state

To confirm the possibility of discriminating the three emotional states, we performed one-way ANOVA on each index. The result showed that the conditions had a significant effect on each index at *p* < 0.001 for the three emotional states (normal, fear/anxiety, and appetitive state): [$\mathrm{F}\left(2, 2757\right)= 565.36, p<0.001$] for swimming velocity, [$\mathrm{F}(2, 2757) = 60.72, p<0.001$] for probability of stopping, [$\mathrm{F}(2, 2757) = 24.85, p<0.001$] for ventilatory signal frequency, [$\mathrm{F}(2, 2757) = 61.19, p<0.001$] for the SD of the swimming velocity, [$\mathrm{F}(2, 2757) = 824.94, p<0.001$] for the SD of the probability of stopping, and [$\mathrm{F}(2, 2757) = 114.68,p<0.001$] for the SD of the ventilatory signal frequency.

**Figure 7:**
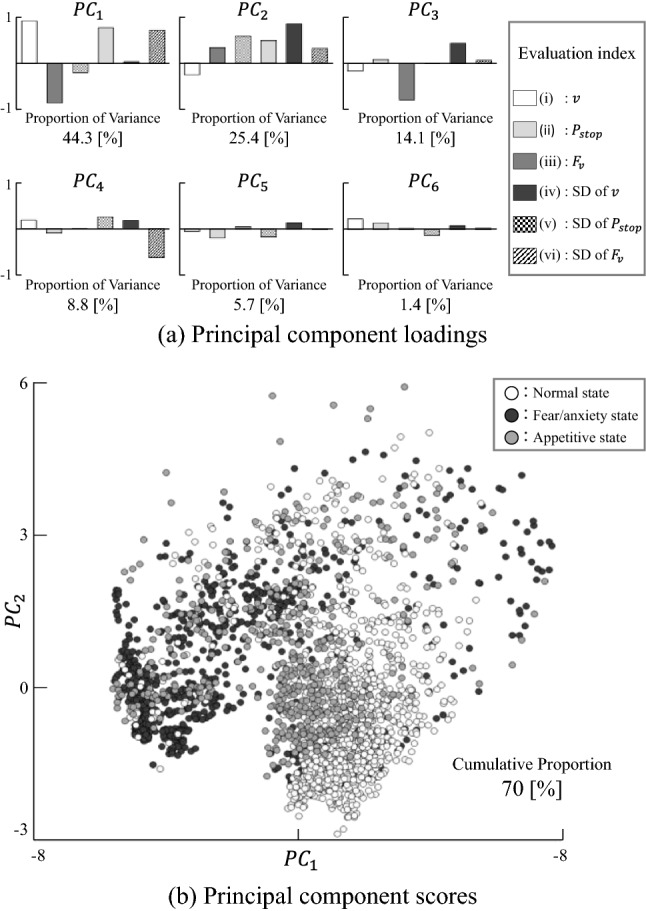
PCA results. The data for the fear/anxiety state are closely grouped in the bottom left-hand portion of the space and are distributed differently from the data for the normal state. The data for the appetitive state are widely distributed throughout the space.

Then, we performed PCA on the six indices. Figure 7a shows the obtained PC loading, and Fig. 7b shows the scatter plot of the first and second PC scores. In the PC space, the fear/anxiety state is relatively closely grouped, whereas the appetitive state is widely dispersed. This suggests that fear/anxiety is directly linked to a perception of crisis and causes uniform behaviours, whereas an appetitive state induces various behaviours that may reflect complex information interpretation in the brain. From the scatter plot of the PC scores, the differences between the normal, fear/anxiety, and appetitive states can be visually confirmed. In addition, we performed one-way ANOVA on each PC. Again, the conditions have a significant effect on each PC at *p* < 0.001 for the three emotional states: [$\mathrm{F}(2, 2757) = 452.03, p<0.001$] for the first PC, [$\mathrm{F}(2, 2757) = 169.95,p<0.001$] for the second PC, [$\mathrm{F}(2, 2757) = 39.57, p<0.001$] for the third PC, [$\mathrm{F}(2, 2757) = 16.08, p<0.001$] for the fourth PC, [$\mathrm{F}(2, 2757) = 98.69, p<0.001$] for the fifth PC, and [$\mathrm{F}(2, 2757) = 23.52, p<0.001$] for the sixth PC.

These results suggest the possibility that the defined indices can capture the differences between the normal, fear/anxiety, and appetitive states. Therefore, we used Fisher's linear discriminant analysis and the probabilistic neural network LLGMN to discriminate the three emotional states. The PCs of the indices constitute the input dimensions of the models. The input dimensions were increased from one to six by adding each PC individually.

**Figure 8:**
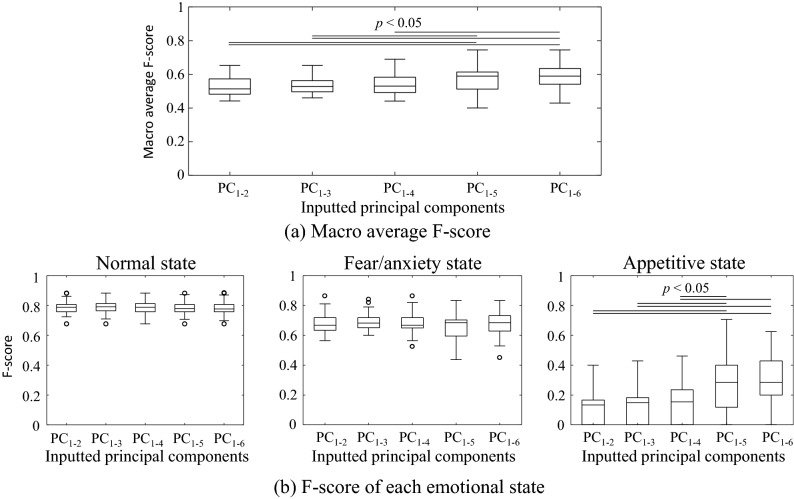
F-scores obtained from the linear discriminant analysis. PC_1−*N*_ denotes that the first through the *N*th PCs were used for discrimination. (**a**) Boxplot of macro average F-scores for all emotional states. (**b**) F-scores of each emotional state. In the macro average and appetitive states, the F-scores significantly increases with the PC used for discrimination.

Figure 8 shows the F-scores obtained from the linear discriminant analysis. Figure 8a shows the macro average F-score, i.e. the average F-score of the three emotional states. Figure 8b shows the F-scores of each emotional state. The significant differences that can be observed in the figure indicate that the discrimination performance increases with the increment in the PC used for discrimination. Specifically, the discrimination for the appetitive state was significantly improved by adding the fifth PC, and thus the overall discrimination performance (macro average F-score) was also increased. These results also indicate that linear discriminant analysis cannot easily classify the appetitive state because its F-scores is in a low range (from approximately 0 to 0.4).

**Figure 9:**
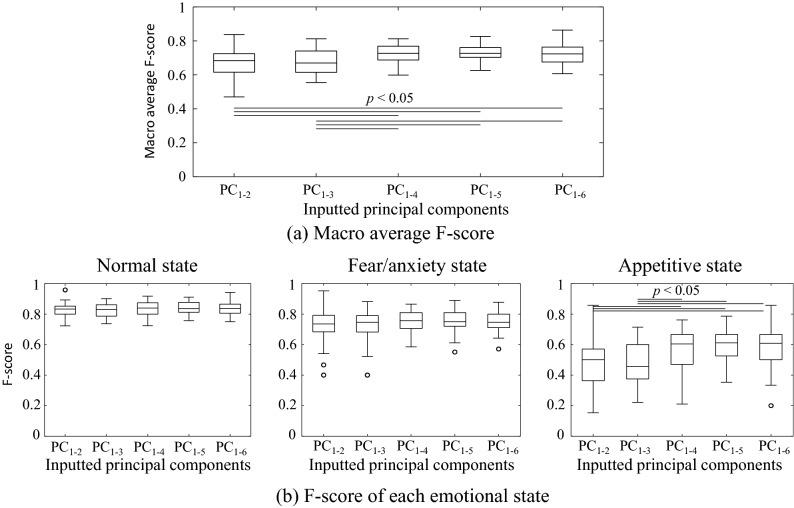
F-scores obtained from LLGMN. (**a**) Boxplot of macro average F-scores for all emotional states. (**b**) F-scores of each emotional state. In the macro average and the appetitive state, the F-scores of PC_1–4_, PC_1–5_, and PC_1–6_ are significantly higher than those of PC_1–2_ and PC_1–3_.

Figure 9 shows the F-scores obtained from the LLGMN, which is a nonlinear classifier involving a statistical structure in an artificial neural network form. The LLGMN yielded the best F-score of 0.84 for the normal state, and yielded F-scores of 0.76 and 0.59 for the fear/anxiety and appetitive states, respectively. The significant difference that can be observed in the figure indicates that the addition of the fourth PC significantly improved the discrimination performance for the appetitive state as well as the macro average F-score. These results show that the appetitive state cannot be separated linearly from the other two emotional states but can be discriminated from the other two states with the nonlinear model. The F-score of the appetitive state increased significantly when the fourth PC was added: up to the third and fourth PCs, the mean macro average F-score were approximately 0.67 and 0.72, respectively. There was no significant improvement in the macro average F-score when the fifth and sixth PCs were added as inputs. For the fourth PC, the PC loading was the largest with respect to the index (vi), which is the SD of the peak frequency of the ventilatory signals (see Fig. 7). The stability of the respiratory rhythm was thus an effective measure for classifying the appetitive state. These results demonstrate the effectiveness of emotional state evaluation when combining ventilatory information with motion information.

**Figure 10:**
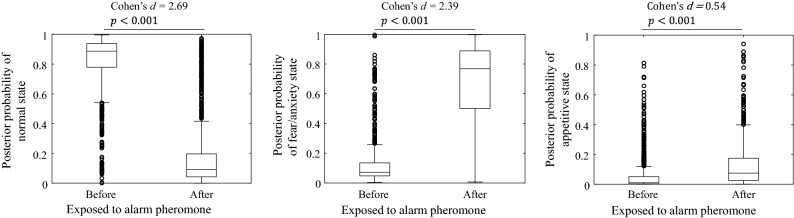
Posterior probability obtained from LLGMN in the alarm pheromone experiment. The plots compare the posterior probabilities of each emotional state between before and after exposure to the alarm pheromone.

**Figure 11:**
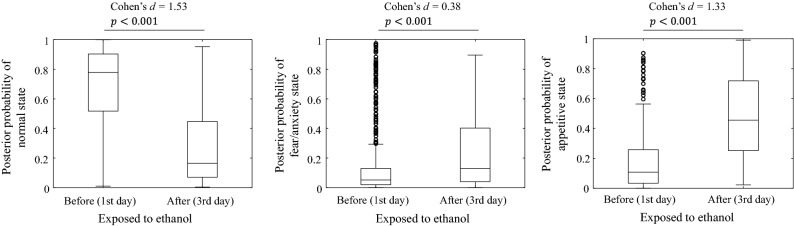
Posterior probability obtained from LLGMN in the ethanol-induced place preference experiment. The plots compare the posterior probabilities of each emotional state between before and after conditioning using ethanol in the 2nd day of the experiment.

Figures 10 and 11 show boxplots of the posterior probabilities for each emotional state obtained from the alarm pheromone and ethanol-induced place preference experiments, respectively. The time-series data of the swimming velocity, respiratory frequency, and posterior probability can be found in Supplementary Information S3. From Fig. 10, it can be seen that according to the Welch's *t*-test, the posterior probability of normal state decreased significantly, and those of the fear/anxiety and appetitive states increased significantly after exposure to the alarm pheromone. Cohen's *d* showed that the effect of the increase in the posterior probability of the fear/anxiety state was larger than that of the appetitive state. In most case, the LLGMN correctly predicted the fear/anxiety state after the fish was exposed to the alarm pheromone. In Fig. 11, after the treatment of the 2nd day, when the fish was exposed to ethanol, the posterior probability of the normal state significantly decreased, and those of the fear/anxiety and appetitive states significantly increased. Cohen's *d* showed that the effect of the increase in the posterior probability of the appetitive state was larger than that of the fear/anxiety state. In other words, in most cases, the LLGMN correctly predicted the appetitive state after the fish was exposed to ethanol. These results indicate that the emotional state could be discriminated by collecting samples of the posterior probabilities and perform a statistical test, which can compensate the instantaneous misclassifications that lowered F-scores.

## Discussion

In this paper, we proposed a method for evaluating the emotional state of fish by estimating the fish position and swimming velocity in real-time. In the experiments conducted in this study, the fear/anxiety state and the appetitive state were induced using an alarm pheromone and ethanol, respectively.

The radar chart indices (see Fig. 6) demonstrate that the indices defined in this study can detect the characteristics of the fear/anxiety state and the appetitive state. In the fear/anxiety state, fish exhibit freezing behaviours. This observation is consistent with those of previous studies^17,18^, as well as those of our previous study^7^, in which we found that the frequency of the ventilatory signal gradually decreased with time after exposure to an alarm pheromone, even when a fish was in the fear/anxiety state (see Fig. 10). This may reflect a decrease in the level of fear/anxiety with time. In the appetitive state, fish exhibits various behaviours, but its ventilation rhythm stabilised with time.

In the ethanol-induced place preference experiment, only about a half of the fish showed place preference after the treatment in the second day (see Fig. 5). This can be caused by the size of the chamber used in the experiment. The dimension of the chamber reported by the original literature^4^ was 52 cm in length, 16.6 cm in width, and 22.9 cm in depth, whereas our chamber was 21 cm in length, 14 cm in width, and 65 cm in depth. As a result, the gap between the dotted area and the white area became small. The chamber was made small and shallow to increase the density of the electrode array and to place the electrode closer to the fish body. This problem could be solved by increasing the size of the chamber by increasing the number of electrodes. It is also important to enable measurement of the ventilatory signals even when the fish are distant from the bottom of the chamber where the electrodes are placed; this aspect will be considered in future works.

By performing PCA and plotting the indices on the first and second PC surface, differences in the distributions of the emotional states can be visually confirmed. This suggests that the emotional state of zebrafish can be expressed on this PC space. The linear and nonlinear discrimination models used allowed the discrimination of the emotional states considered (see Figs. 8 and 9). The SD of the peak frequency of the ventilatory signals was found to be an effective measure for discriminating the appetitive state from the other two emotional states. In the behavioural catalogue^6^, ventilatory information was associated with fear/anxiety states; however, the fact that discrimination performance was improved by adding a PC with the highest loading of ventilation information indicates that the ventilatory information is also associated with the appetitive state. This finding implies that focusing on ventilatory information may allow us to detect a greater variety of emotional states.

Nevertheless, the discrimination performance for the appetitive state is insufficient for experimental application. This is because we discriminated the emotional states every 5 s using the average values. The radar plots before and after exposure to the alarm pheromone and ethanol are very similar, and only the degrees of change are different (see Figs. 4 and 6). In the scatter plot of the principal component scores (see Fig. 7b), the plot points move from the normal state to the upper left in the order of the appetitive state and fear/anxiety state. Therefore, the neural network may discriminate emotional states mainly according to the degree of changes along this axis toward the upper left in the PC space. This raises the importance of considering time trajectories of indices to improve the future discrimination performance. The values of the indices under the appetitive state spread widely on the surface of the first and second PCs, and mostly overlapped with either normal or fear/anxiety states (see Fig. 7). This observation indicates that the values of the indices may largely vary over time. Therefore, the introduction of machine learning techniques that can learn the time trajectory, such as recurrent neural network, could improve the discrimination performance on the appetitive state and thus the total discrimination performance as well.

Apart from ethanol, there are various chemical substances that can be used to induce appetitive states. Caffeine, morphine, and cocaine have been shown to induce preference-exhibiting behaviours as well^19,20^. Therefore, it is possible that the appetitive state could be evaluated in more detail by changing the chemical substance used. We found that the fear/anxiety and appetitive states increase and lower the frequency of ventilation, respectively. Thus, the mechanisms for controlling the ventilatory rhythm and its relationship to the emotional states should be investigated further, which may lead to more effective indices for evaluating emotional states.

The proposed system can analyse fish movement in a two-dimensional space but not in three-dimensional space. This limitation can be addressed by placing electrodes on the side of the aquarium and estimating the position and velocity using the method described in this paper. In addition, the proposed system is not suitable for experiments with more than one fish. Zebrafish have a habit of exhibiting group behaviour; thus, the emotional state of zebrafish can be estimated by measuring their interactions. We intend to attempt this by introducing blind source separation technology, by means of which we will separate the ventilatory signals measured for multiple animals using the characteristics of the frequency components of each fish, and we will estimate their movements. Further, it is challenging to track the movement of zebrafish larvae, which often lack pigment, using a video camera system. The proposed system may be applicable for the tracking of larvae but may require more sensitive amplification and denoising techniques. Therefore, in future works, we will attempt the simultaneous measurement of ventilatory signals and movements.

## Supplementary Information

Supplementary Information.
