# Supplementary material for: Measurement of emotional states of zebrafish through integrated analysis of motion and respiration using bioelectric signals
Source: Sci Rep. 2021 Jan 8;11:187. doi: 10.1038/s41598-020-80578-6 (PMC7794612; doi:10.1038/s41598-020-80578-6)
Supplement: Supplementary file 1 — Supplementary Information. [file 41598_2020_80578_MOESM1_ESM.docx]

**Measurement of Emotional States of Zebrafish through Integrated Analysis of Motion and Respiration Using Bioelectric Signals**

Zu Soh^1*^, Motoki Matsuno^2^, Masayuki Yoshida^3^, and Toshio Tsuji^1*^

^1^ Graduate School of Advanced Science and Engineering, Hiroshima University, Higashi-Hiroshima, 739-8527, Japan

^2^ Graduate School of Engineering, Hiroshima University, Higashi-Hiroshima, 739-8527, Japan

^3^ Graduate School of Integrated Sciences for Life, Hiroshima University, Higashi-Hiroshima, 739-8527, Japan

E-mail: sozu@bsys.hiroshima-u.ac.jp (ZS), tsuji@bsys.hiroshima-u.ac.jp (TT)

**Supplemental Information**

#

# S1. Ventilatory signals

Fish are surrounded by an electrical field^1^. Equipotential lines are dense in the area between the gill and anus. The osmotic pressure of the fish is considered integral to the generation of this potential. When the operculum opens, ions migrate inside and outside the body in biological membranes^2^, and the movement of these ions is restricted when the operculum closes. This phenomenon generates an electrical potential that is synchronized with ventilation. These bioelectrical signals are called ventilatory signals^1^. Measuring the ventilatory signals in fish first became possible when Gruber *et al*. developed a noise elimination amplifier in 1977^3^, and research using ventilatory signals began soon afterward. Our research group has proposed bioassay systems that use the ventilatory signals of medaka and zebrafish and has confirmed that ventilatory signals can be detected in the frequency band of approximately 1–10 Hz^4,5^. Ventilation is controlled by the autonomic rhythm generated by the respiratory centre, which exhibits acute responses to changes in the chemical environment^6^. In addition, because the contraction and relaxation of gill vessels are adjusted according to the state of the sympathetic nerve, ventilatory signals are considered to be indirectly influenced by autonomic nerves^1,7^. When a fish expresses fear and anxiety, its ventilation, heart rate, and blood pressure change^8^. Consequently, ventilatory signals contain information regarding such emotions. For these reasons, we focused on ventilatory signals in this study.

# S2. Log-linearised Gaussian Mixture Network (LLGMN)

Figure S1 shows the structure of the LLGMN used in this study. The LLGMN calculates the posterior probability $P(c,m|\boldsymbol{x})$ of class $c(c=1,\ldots, C)$ from the input feature vector $\boldsymbol{x}$. In Fig. S1, $⊘$ represents a linear unit to input signals. To represent the parameters included in a normalised distribution corresponding to each component of a Gaussian mixture model (GMM) as weight coefficients of the LLGMN, the input vectors$\boldsymbol{x} \in\mathbb{R}^{D}$ are nonlinearly converted as follows:

|  | $\boldsymbol{X}\boldsymbol{=}\left[ \boldsymbol{1,}\boldsymbol{x}^{\boldsymbol{T}}\boldsymbol{,}\boldsymbol{x}_{\boldsymbol{1}}^{\boldsymbol{2}}\boldsymbol{,}\boldsymbol{x}_{\boldsymbol{1}}\boldsymbol{x}_{\boldsymbol{2}}\boldsymbol{,\ldots,}\boldsymbol{x}_{\boldsymbol{1}}\boldsymbol{x}_{\boldsymbol{D}}\boldsymbol{,}\boldsymbol{x}_{\boldsymbol{2}}^{\boldsymbol{2}}\boldsymbol{,}\boldsymbol{x}_{\boldsymbol{2}}\boldsymbol{x}_{\boldsymbol{3}}\boldsymbol{,\ldots,}\boldsymbol{x}_{\boldsymbol{2}}\boldsymbol{x}_{\boldsymbol{D}}\boldsymbol{,\ldots,}\boldsymbol{x}_{\boldsymbol{D}}^{\boldsymbol{2}} \right]^{\boldsymbol{T}}$. | (B.1) |
| --- | --- | --- |

The first layer of the LLGMN consists of $H$ units corresponding to the dimension of $\boldsymbol{X}$, and the *h*^th^ unit outputs the value of the *h*^th^ element $X_{h}\in X$ as follows:

|  | ${{}^{\boldsymbol{(1)}}\boldsymbol{I}}_{\boldsymbol{h}}\boldsymbol{=}\boldsymbol{X}_{\boldsymbol{h}}$, | (B.2) |
| --- | --- | --- |
|  | ${{}^{\boldsymbol{(1)}}\boldsymbol{O}}_{\boldsymbol{h}}\boldsymbol{=}{{}^{\boldsymbol{(1)}}\boldsymbol{I}}_{\boldsymbol{h}}$, | (B.3) |

where ${{}^{(1)}I}_{h}$ and ${{}^{(1)}O}_{h}$ are the input and output of the $h$^th^ unit, respectively.

The second layer consists of $\sum_{c=1}^{C} M_{c}$ units, where $C$ and $M_{c}$ are the number of classes and the number of components included in class $c$, respectively. Each unit receives the output of the first layer, weighted by the coefficient $w_{h}^{(c,m)} (m=1, \ldots,M_{c})$, which corresponds to the parameters included in the GMM. The input${{}^{(2)}I}_{c,m}$ and the output ${{}^{(2)}O}_{c,m}$ are defined as follows:

|  | ${{}^{\boldsymbol{(2)}}\boldsymbol{I}}_{\boldsymbol{c,m}}\boldsymbol{=}\sum_{\boldsymbol{h=1}}^{\boldsymbol{H}} {{}^{\boldsymbol{(1)}}\boldsymbol{O}}_{\boldsymbol{h}}\boldsymbol{w}_{\boldsymbol{h}}^{\boldsymbol{(c,m)}}$, | (B.4) |
| --- | --- | --- |
|  | ${{}^{\boldsymbol{(2)}}\boldsymbol{O}}_{\boldsymbol{c,m}}\boldsymbol{=}\frac{\mathbf{exp}\left[ {{}^{\boldsymbol{(2)}}\boldsymbol{I}}_{\boldsymbol{c,m}} \right]}{\sum_{\boldsymbol{c}^{\boldsymbol{'}}\boldsymbol{=1}}^{\boldsymbol{C}} \sum_{\boldsymbol{m}^{\boldsymbol{'}}\boldsymbol{=1}}^{\boldsymbol{M}_{\boldsymbol{c}^{\boldsymbol{'}}}} \mathbf{exp}\left[ {{}^{\boldsymbol{(2)}}\boldsymbol{I}}_{\boldsymbol{c}^{\boldsymbol{'}}\boldsymbol{,}\boldsymbol{m}^{\boldsymbol{'}}} \right]}$. | (B.5) |

The third layer consists of two units, and unit $c$ integrates the output of $M_{c}$ units in the second layer. The relationship between ${{}^{(3)}I}_{h}$ and ${{}^{(3)}O}_{h}$ is defined as follows:

|  | ${{}^{\boldsymbol{(3)}}\boldsymbol{I}}_{\boldsymbol{c}}\boldsymbol{=}\sum_{\boldsymbol{m=1}}^{\boldsymbol{M}_{\boldsymbol{c}}} {{}^{\boldsymbol{(2)}}\boldsymbol{O}}_{\boldsymbol{c,m}}$, | (B.6) |
| --- | --- | --- |
|  | ${{}^{\boldsymbol{(3)}}\boldsymbol{O}}_{\boldsymbol{c}}\boldsymbol{=}{{}^{\boldsymbol{(3)}}\boldsymbol{I}}_{\boldsymbol{c}}$. | (B.7) |

The output ${{}^{\boldsymbol{(3)}}\boldsymbol{O}}_{\boldsymbol{c}}$ corresponds to the posterior probability for the inputted feature vectors $\boldsymbol{x,}$ calculated based on Bayes' theorem.

Provided with the teacher vector $\boldsymbol{T}^{(n)}={[T_{1}^{\left( n \right)},\ldots,T_{c}^{\left( n \right)},\ldots,T_{C}^{\left( n \right)}]}^{T}$ for the *n*^th^ input vector $\boldsymbol{x}^{\left( n \right)}(n=1,\ldots,N)$, supervised learning of the LLGMN is performed to minimize the network energy function, defined based on Kullback–Leibler information as follows:


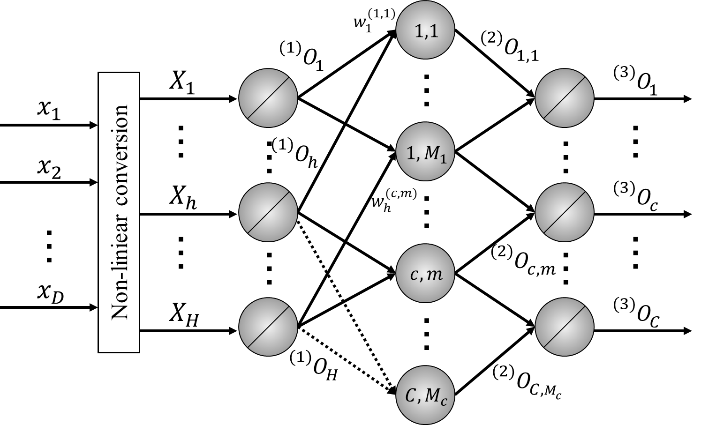


**FIG. S1**. Structure of the log-linearised Gaussian mixture network (LLGMN).

**FIG. S1**. Electrical field distribution around carp, as recorded by Asano and Hanyu^20^ (revised from literature^10^). The isoelectric lines became dense around the gills and anus (indicated by arrows).

|  | $\boldsymbol{J}\mathbf{=-}\sum_{\boldsymbol{n=1}}^{\boldsymbol{N}} \boldsymbol{J}_{\boldsymbol{n}}\boldsymbol{=}\mathbf{-}\sum_{\boldsymbol{n=1}}^{\boldsymbol{N}} \sum_{\boldsymbol{c=1}}^{\boldsymbol{C}} \boldsymbol{T}_{\boldsymbol{c}}^{\left( \boldsymbol{n} \right)}\mathbf{log}^{\boldsymbol{(3)}}\boldsymbol{O}_{\boldsymbol{c}}^{\left( \boldsymbol{n} \right)}$. | (B.8) |
| --- | --- | --- |

The weight modification $\Delta w_{h}^{(c,m)}$ is defined as follows:

|  | $\boldsymbol{\Delta}\boldsymbol{w}_{\boldsymbol{h}}^{\boldsymbol{(c,m)}}\boldsymbol{=}\mathbf{-}\boldsymbol{\eta}\sum_{\boldsymbol{n=1}}^{\boldsymbol{N}} \sum_{\boldsymbol{c=1}}^{\boldsymbol{C}} \frac{\boldsymbol{\partial}\boldsymbol{J}_{\boldsymbol{n}}}{\boldsymbol{\partial}\boldsymbol{w}_{\boldsymbol{h}}^{\boldsymbol{(c,m)}}}$, | (B.9) |
| --- | --- | --- |
|  | $\frac{\boldsymbol{\partial}\boldsymbol{J}_{\boldsymbol{n}}}{\boldsymbol{\partial}\boldsymbol{w}_{\boldsymbol{h}}^{\left( \boldsymbol{c,m} \right)}}\boldsymbol{=}\frac{\boldsymbol{\partial}}{\boldsymbol{\partial}\boldsymbol{w}_{\boldsymbol{h}}^{\left( \boldsymbol{c,m} \right)}}\left( \sum_{\boldsymbol{c=1}}^{\boldsymbol{C}} \boldsymbol{T}_{\boldsymbol{c}}^{\left( \boldsymbol{n} \right)}\mathbf{log}^{\left( \boldsymbol{3} \right)}\boldsymbol{O}_{\boldsymbol{c}}^{\left( \boldsymbol{n} \right)} \right)$, |  |
|  | $\boldsymbol{=}\left( {}^{\boldsymbol{(2)}}\boldsymbol{O}_{\boldsymbol{c}}^{\left( \boldsymbol{n} \right)}\boldsymbol{-}\frac{{}^{\boldsymbol{(2)}}\boldsymbol{O}_{\boldsymbol{c}}^{\left( \boldsymbol{n} \right)}}{{}^{\boldsymbol{(3)}}\boldsymbol{O}_{\boldsymbol{c}}^{\left( \boldsymbol{n} \right)}}\boldsymbol{T}_{\boldsymbol{c}}^{\left( \boldsymbol{n} \right)} \right)\boldsymbol{X}_{\boldsymbol{h}}^{\left( \boldsymbol{n} \right)}$, | (B.10) |

where $\boldsymbol{\eta}$> 0 is the learning rate. The weight coefficients are iteratively modified so that the LLGMN learns the distribution of the teacher vector on the feature vectors for the purpose of calculating the posterior probability.

# S3. Time series data of swimming velocity, ventilatory signal frequency, and posterior probabilities yielded by LLGMN

Figures S2-1 and S2-2 show time series data for the posterior probability of the fear/anxiety state and that of the ethanol-induced place preference state, respectively, obtained using the LLGMN. Figure S2-1 shows that the posterior probability of fear/anxiety increased after exposure to the alarm pheromone for all individuals. The figure shows that the a posteriori probability increased when the velocity decreased or the respiratory frequency increased, and it was relatively easy to visually discriminate the fear/anxiety state from the other two states. Figure S2-2 shows that the posterior probability of the ethanol-induced place preference state. The figure shows that the a posteriori probability of the pleasure state increased when the respiratory frequency was relatively stable, but the pleasure state was not as easy to discriminate as to the fear/anxiety state. This is why the nonlinear model is required to discriminate the ethanol-induced place preference state from the other two emotional states.

| 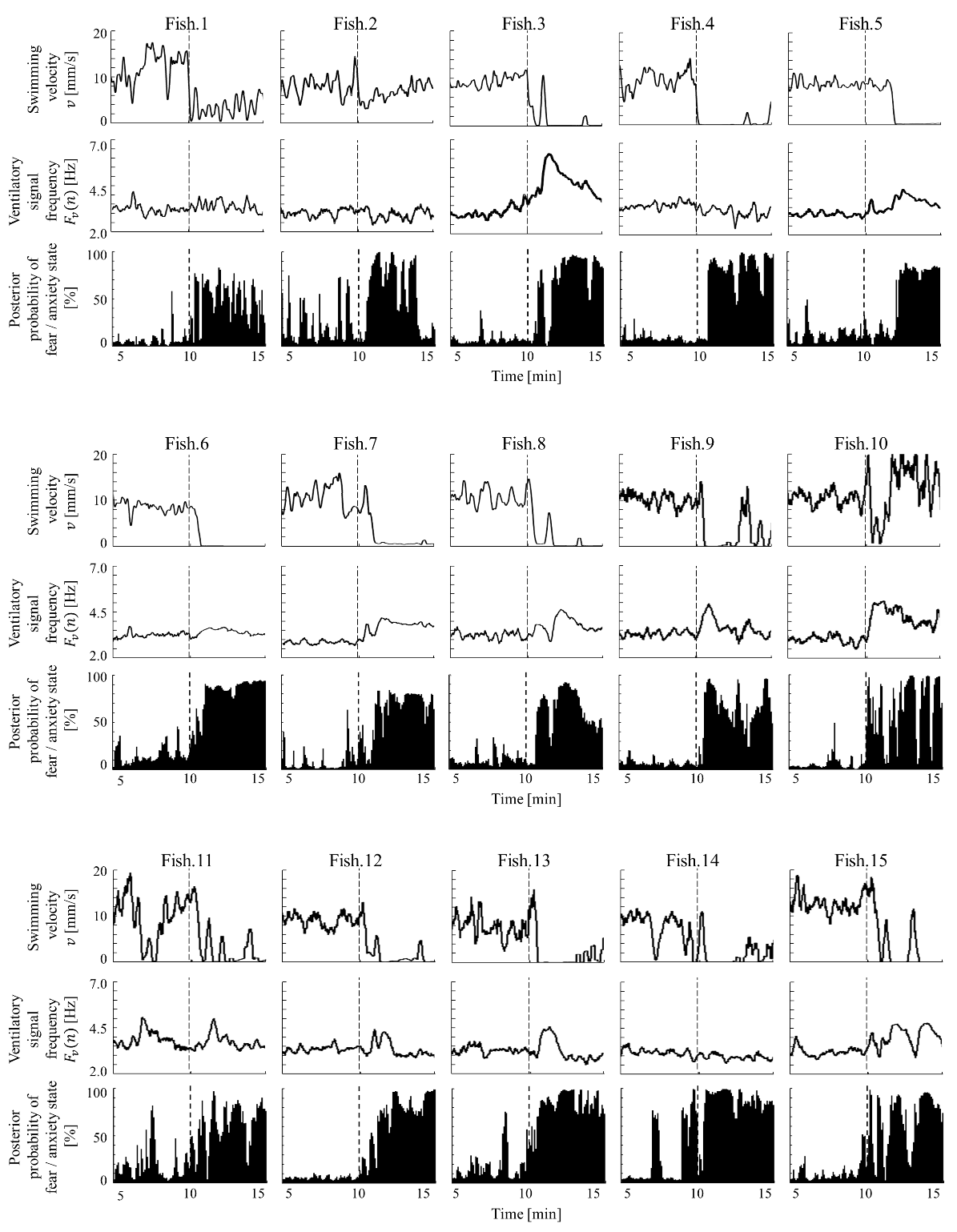  **FIG. S2-1**. Time series data for the evaluation indices for all fifteen individuals in experiments conducted to induce the fear/anxiety state. The fish were exposed to the alarm substance at 10 min, as indicated by the dotted vertical lines. After exposure, the posterior probability of the fear/anxiety state was increased. |
| --- |
| 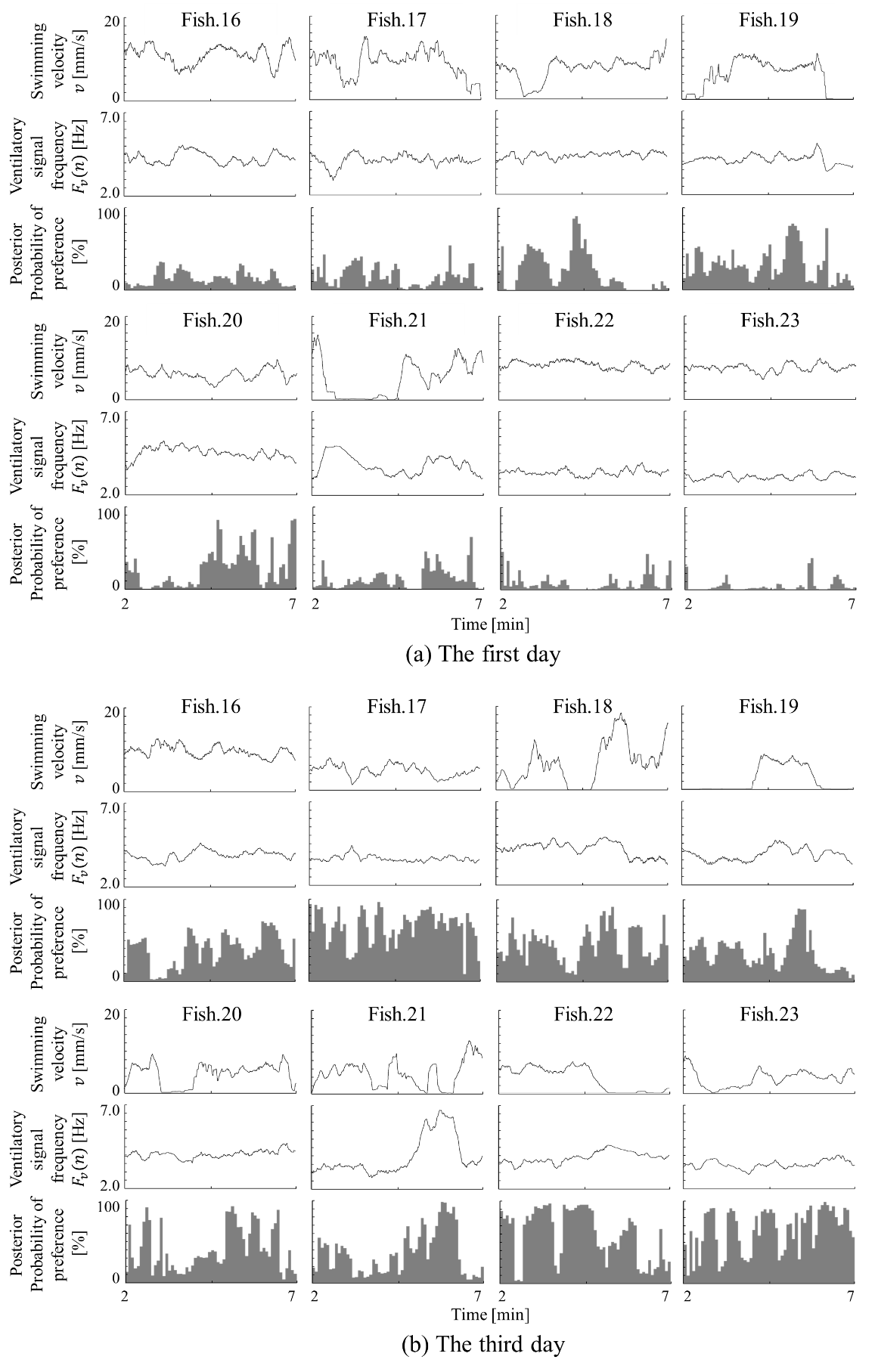  **FIG. S2-2**. Time series data of the indices for all eight individuals in experiments conducted to induce the pleasure state. The fish were exposed to ethanol on the second day. After exposure, the posterior probability of the pleasure state was increased. |

# References

**1.** Asano M, Hanyu I. Fish Behaviour and Bioelectricity, Biology of Migrants. Japan Scientific Societies Press, Tokyo, Japan, 1987, pp. 181-195.

**2.** Iwata S, Hirano T. Osmotic Pressure Mechanism, Fish Physiology (in Japanese). Koseisha Koseikaku, Tokyo, Japan, 1991.

**3.** Gruber D, Cairns J, Dickson KL, Hummel R, Maciorowski A, Schalie WHVD. An Inexpensive, Noise-Immune Amplifier Designed for Computer Monitoring of Ventilatory Movements of Fish and Other Biological Events. Trans Amer Fish Soc 1977;106(5):497-499.

**4.** Terawaki M, Hirano A, Soh Z, Tsuji T. Unconstrained and Noninvasive Measurement of Bioelectric Signals from Small Fish. Artif Life Robot 2009;14:728-733.

**5.** Kitayama S, Soh Z, Hirano A, Tsuji T, Takiguchi N, Ohtake H. Unconstrained and Noninvasive Measurement of Swimming Behaviour of Small Fish Based on Ventilatory Signals. SICE 2012;48:151-158.

**6.** Ozaki H. Fishes Physiologic Course. Ryokushoboh 1970;43. [in Japanese]

**7.** Jonz MG, Zaccone G. Nervous Control of the Gills. Acta Histochem 2009;111:207-216.

**8.** Yoshida M, Hirano R, Shima T. A Novel Method for Monitoring Cardiac Activity in Fish. Zool Soc Japan 2009;26:356-361.
